# Developing a Round-Robin Module For The Integration Of Consensus Standards In a BME Course Using a Custom Tensile Testing Device

**DOI:** 10.1007/s43683-025-00200-x

**Published:** 2025-10-07

**Authors:** Chara Nunnally, Adrian P. Defante, Michael G. Browne, Anthony E. Felder

**Affiliations:** 1 Richard and Loan Hill Department of Biomedical Engineering, University of Illinois Chicago, Chicago, IL USA; 2 Hollister Inc, Libertyville, IL USA

**Keywords:** Biomedical engineering, Engineering education, Round-Robin testing, Curriculum, Tensile testing, Material testing

## Abstract

**Purpose:**

Proficiency with consensus standards is essential for biomedical engineers to develop effective, safe, and compliant medical devices. Here, we describe a novel, standards-based module that enhances student ability to interpret, apply, and revise consensus standards through round-robin testing.

**Methods:**

A hands-on learning module was designed and implemented in an upper-level biomedical engineering course. The curriculum incorporated the use of a custom-designed tensile testing device alongside a mock standard to introduce students to protocol development, standards revision, and real-world challenges in testing variability. Eight student teams conducted round-robin testing using devices configured with deliberate adulterations. Learning objectives (LO) include (1) defining round-robin testing, (2) interpreting a consensus standard, and (3) revising a consensus standard. Assessment included a Standard Revision Report and a post-module survey.

**Results:**

From the post-module survey, students were only somewhat able to define round-robin testing (LO1; average score of 0.4/1). From the Standard Revision Report, teams reliably identified elements from the mock standard to apply for their own tensile testing (LO2; average score of 2.5/3). Also from the Report, teams reliably revised the mock standard to address the adulterations they found (LO3; average score 1.5/2). After the module, students reported confidence in extracting requirements from standards, applying them to verification testing, and identifying potential limitations in testing protocols. Moreover, students found the activity effective for increasing their confidence in preparing them for industry applications, though some suggested extending the module duration and improving instructional clarity for increased effectiveness.

**Conclusion:**

This study describes the development and implementation of a standards-based module in biomedical engineering. Ultimately, students engaged in higher-order problem-solving and improved their understanding of standards implementation, testing variability, and collaborative verification processes. The findings suggest that this curriculum model could be expanded across engineering disciplines to enhance workforce preparedness in quality engineering and R&D roles.

**Supplementary Information:**

The online version contains supplementary material available at 10.1007/s43683-025-00200-x.

## Introduction

In many product development industries, consensus standards serve as guidelines to ensure consistency and reliability between like-products, while also helping promote the safety and efficacy of the technology or processes. These standards help build customer confidence by establishing the criteria by which a process is managed, a technology is designed and manufactured, or a technique is performed [1]. Moreover, standards are recognized by industry professionals as a necessity for employment [2, 3]. Over the past decade, significant strides have been made in integrating consensus standards education into engineering curricula across various disciplines. In support of standards education, universities have partnered with the ANSI Outreach Program to promote the incorporation of standards-related information in engineering curricula [4]. Moreover, many university libraries have increased access to standards, recognizing their importance in engineering practice and helping facilitate the design of novel educational programming [5]. Consequentially, new efforts have been marked by a blend of lecture-based instruction, in-class discussions, and hands-on activities in both asynchronous and synchronous modes. Mechanical and Electrical Engineering programs have made substantial progress in incorporating standards education, with many departments focusing on specific industry needs to prepare their students to enter the workforce [6, 7]. Another example, from the University of Tennessee and Case Western Reserve University, compiled known free and paid resources around teaching of standards and provided their own universal standards module aimed at lower-level undergraduate courses [8].

Instructional content for consensus standards is most commonly included in capstone design. Indeed, the Accreditation Board for Engineering and Technology (ABET) Criterion 5 specifies that credentialed engineering curriculum must include “a culminating major engineering design experience that (1) incorporates appropriate engineering standards and multiple constraints, and (2) is based on the knowledge and skills acquired in earlier course work” [9]. Standards have also been incorporated into other upper-level courses to foster a deeper understanding of the regulatory frameworks that govern engineering practice. For example, Mechanical Engineering students enrolled in a 400-level course at Purdue University engaged in projects where they were tasked with identifying and applying relevant technical standards, including those related to manufacturing, safety, and Quality Management Standards when designing “everyday items” [6]. Some courses involve more advanced tasks, such as students creating case studies, analyzing and refining standard operating procedures (SOPs), or designing systems that meet specific industry standards [6, 7]. This approach not only improved students’ understanding of standards but also enhanced their problem-solving abilities, aligning closely with the core responsibilities of various quality professionals.

However, there is limited published information on consensus standards education within biomedical engineering (BME). Standards education is critical in the field of BME as graduates often enter jobs with connections to quality assurance, regulatory compliance, and product development geared toward promoting safety and effectiveness. Indeed, most professionals in the medical engineering industry agree that there is need for engineers to understand the fundamentals of standards development and how standards contribute to alignment between departments such as Research & Development and Quality [3, 10]. The BME departments at the University of Delaware, the University of Michigan, and Milwaukee School of Engineering have aimed to address this deficiency. Studies conducted from these departments support the idea that the integration of consensus standards into the BME curriculum early in a students’ college career provides substantial benefits [5, 11, 12]. Specifically, students whose first-year courses incorporated consensus standards completed their senior design course with a greater functional understanding of standards than students who did not gain exposure to consensus standards until their upper-level courses. Further, LaMack et al. found that early, structured exposure to professional topics leads to measurable improvements in student preparedness for industry-facing roles [13]. However, in general, standards curricula tend to focus on the sourcing of standards, which reflects lower levels of Bloom’s Taxonomy such as “Remember” and “Understand,” while few engage higher levels such as “Analyze,” “Evaluate,” or “Create.” Indeed, engagement with consensus standards in industry has the potential to reasonably mirror Bloom’s Taxonomy. Unfortunately, with some employers, long-ingrained processes (e.g., bookkeeping, training, protocols) may discourage higher level engagement with Bloom’s Taxonomy. However, with others, entry-level engineering roles tend to engage with lower levels of Bloom’s Taxonomy (e.g., using a standard for testing) and senior-level engineering roles may engage with higher levels of Bloom’s Taxonomy (e.g., serve on international standards committees). As a result, greater competency with consensus standards may help graduates assume senior-level engineering industry roles earlier in their careers. Therefore, there is a need for curricular innovation in BME education that not only introduces students to standards but also equips them with the skills to demonstrate proficiency at more advanced levels.

Our recent work-in-progress paper described the development of several standards education modules across all levels of Bloom’s Taxonomy [14]. Here, we describe a curricular module for the revision of consensus standards by incorporating round-robin testing in an in-class activity (module 3 from Ref. [14]). Round-robin testing is an experimental methodology to determine reproducibility of a testing protocol by testing it independently, multiple times [15]. This methodology is useful in evaluating the performance of measurement methods by highlighting the variability of results when the testing is performed by different participants or under differing conditions. For this curricular module, we generated a mock standard entitled *ASTM BME410-24* based on ASTM D412-16 [16]. This mock standard describes testing methodology to characterize the Young’s Modulus of a polyurethane material using a tensile testing device. Moreover, to emphasize discrepancies in testing between groups that can decrease reproducibility during round-robin testing, six of eight of our tensile testing devices received one deliberate adulteration each. The learning objectives of this module include the ability for students to do the following:

1. Understand and define the round-robin standards assessment process (Bloom’s Taxonomy levels 1 and 2; assessed from the post-module survey).
2. Interpret and apply a consensus standard in various use cases (Bloom’s Taxonomy levels 3 and 4; assessed from the Standard Revision Report).
3. Collaboratively revise a consensus standard (Bloom’s Taxonomy levels 5 and 6; assessed from the Standard Revision Report).

Through achieving these learning objectives, this module is intended to prepare BME graduates to more adeptly use consensus standards in industry roles.

## Methods

This research study was identified as exempt by an institutional review board at University of Illinois Chicago (STUDY2025-0353). Ultimately, eight student groups conducted round-robin testing with custom tensile testing devices. To reinforce their understanding, students were required to complete several deliverables including group submissions of a Tensile Testing Worksheet as well as a Standard Revisions Report, and an survey probing module outcomes.

### Requisite Student Knowledge and Course Design

This curricular module was designed and implemented at the University of Illinois Chicago Richard and Loan Hill Department of Biomedical Engineering in a high-throughput elective course focused on FDA regulatory systems and performance standards associated with medical devices (BME 410: FDA and ISO Standards for the Design and Manufacture of Medical Devices). This class is intended for upper-level undergraduate and graduate students, though this iteration happened to have only undergraduate students enrolled.

BME 410 was redesigned between August 2023 and May 2025 with a goal of more fully preparing students for careers in medical devices and with the assistance of funding from the National Institute of Standards and Technology. Prior to the module described in this report, course content includes introductions to U.S. federal regulations (i.e., Code of Federal Regulations), regulatory bodies (i.e., FDA), standards organizations (e.g., ISO, ASTM), and pathways to FDA approval (i.e., pre-market notification vs. pre-market approval). The curriculum also provides students with the skills necessary to source both performance and testing consensus standards in collaboration with the university library leveraging tools such as ASTM Compass. Lecture content describes the difference between performance and testing standards as well as that between applying a standard in general use (e.g., ISO 13485 assigns a quality process to an organization) and customized use cases (e.g., selection of necessary ISO 10993 biocompatibility standards for an ophthalmologic therapy device). After instructing students on the fundamentals of quantifiable product requirements, a group deliverable challenges students to extract requirements from performance standards related to a specific medical device. Lastly, to alleviate issues with prerequisites, a single class period was allocated to provide basic mechanics prior to beginning the module. A lecture with embedded examples gave a refresher on stress, strain, Hooke’s Law, and elastic modulus calculations to ensure student ability to perform calculations appropriately during the module. Example problems during class-time were used to specifically guide the calculation of Young’s modulus using an in-series elastic tensile testing system such as the one we developed for the module.

The remainder of the course includes deep dives into Class II device predicate selections and substantial equivalency comparisons along with a final New Standard Development activity and report, which aim to reach the highest levels of Bloom’s taxonomy through creation of new content. Details of this programming and the entire curriculum will be published in a future report. Lastly, in tandem with the aforementioned course content, students are also assigned individual iterative submissions of a mock 510(k) using the FDA’s eSTAR editable PDF program allowing them to incorporate learnings from the course into successive 510(k) drafts.

### Testing Standard

Students conducted tensile testing using the mock standard entitled *ASTM BME410-24* based on ASTM D412-16. Briefly, the original standard provides thorough methods for users to test vulcanized rubber to failure to determine key material properties. To facilitate the educational objectives related to discrepancy in results during round-robin testing, the standard was modified from the original version for class testing purposes. The mock standard maintained the precision and bias section though it did not affect the activity. Due to ASTM copyright requirements, the modified standard cannot be published in the current report, but relevant changes are listed:

- Revised scope of the standard from vulcanized thermoset rubbers generalized to thermoplastics.
- Revised safety statement to defer to safety protocols in educational settings as prescribed by the instructor.
- Omission of some referenced standards.
- Omissions and revisions of terminology to tailor content to the specific goals of the learning module.
- Omissions and revisions of apparatus section to address limitations of testing in a classroom with the customized tensile testing device.
- Omissions and revisions of specimen section to address limitations of sample size and preparation when testing is carried out in educational setting.
- Omission of references to dynamometers in the calibration section and revising content to encourage validation of testing apparatus.
- Omission of preconditioning and revisions to separation rate in the test procedure section due to time limitations of the class period.
- Revision of content in the Report section to better align with deliverables for the class.

### Tensile Testing Device

**Fig. 1:**
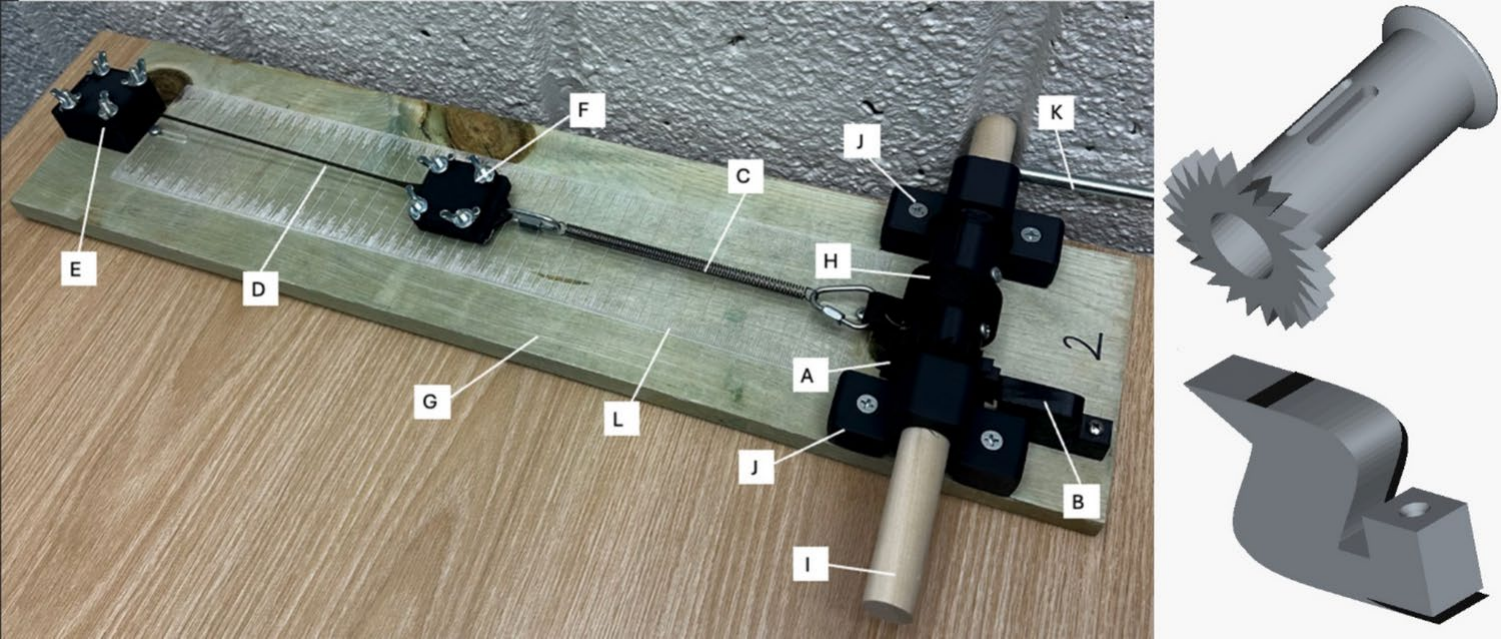
(Left) Custom Tensile Testing Device is shown with labeled components. Component A: Ratcheting Spindle; Component B: Pawl; Component C: Spring; Component D: Material Sample; Component E: Base Clamping Plate; Component F: Floating Clamping Plate; Component G: Wooden Board; Component H: Strapping; Component I: Wooden Dowel Rod; Component J: Dowel Mounts; Component K: Crank Handle; Component L: Etched Ruler. (Right) Computer-Aided Design of the ratcheting spindle (component A) and pawl (component B) are shown on top and bottom, respectively

The tensile testing device was designed using a customized ratcheting spindle (Fig 1, Component A) and pawl (Fig 1, Component B) mechanism to linearly stretch, in series, a spring (Fig 1, Component C) and test sample (Fig 1, Component D). Grooved clamping plates (Fig 1, Components E, F) secure the test sample at both ends to help prevent slippage during testing. Tightening wingnuts are positioned at the corners of both clamping plates, allowing for uniform clamping pressure by hand. One set of grooved clamping plates is fixed to a wooden board substrate (Fig 1, Component G) and the second set of grooved plates is attached, in series, with the spring. The opposite end of the spring is connected to a length of strapping (Fig 1, component H), which wraps around the Ratcheting spindle secured to a wooden dowel rod (Fig 1, Component I) supported by dowel mounts (Fig 1, Component J) affixed to the wooden base. In use, as the crank handle (Fig 1, Component K) is rotated, the spindle induces the ratcheting mechanism against the pawl, drawing the strapping around the spindle and stretching both the spring and testing sample in series. This configuration allows for controlled tension during testing to measure the elastic modulus of the testing specimen. A durable, inelastic strapping material was chosen to minimize fabric strain under load. The base substrate of the device, a 1″ × 8″ 30″ pine board, provides a sturdy platform for the assembly, and its modular design allows for easy adjustments to components. Lastly, an acrylic board etched with graduated markings (Fig 1, Component L) was affixed to the base of the board to facilitate accurate elongation measurements. Test samples were laser cut from both 40A and 60A 1/16″ polyurethane rubber sheets (McMaster-Carr, Illinois, USA) in dumbbell shapes using dimensions corresponding to die c from ASTM D412-16.

The device was designed to facilitate flexible adoption of the curricular module thus the components can be 3D printed on standard fused deposition modeling printers, laser cut, or purchased from common hardware suppliers. All components were designed using SolidWorks (Educational Version, 2023). The spindle, ratchet, and clamping plates were printed using PLA filament, whereas the pawl was printed with flexible TPU to allow for improved flexibility. A complete list of components and their sourcing is available in the supplemental materials (Supplemental Materials Document 1). All 3D print files are publicly available in STL format at the link: https://uofi.box.com/s/476ibec32ysju4jfg5ny3d2pv49hwyxu.

### Calculation of Elastic Modulus

Given the testing sample and spring are in series, force (F) is equivalent between elements and stress (σ) is presumed equivalent for comparable cross-sectional areas. Thus, the elastic modulus of the sample can be calculated using Hooke’s Law and the elastic stress-strain relationship, as shown in the following equation.

$$\sigma =\frac{F}{A}=\frac{kx}{A}=E{\varepsilon }_{\mathrm{sample}}.$$

Here, “*E*” denotes the Young’s Modulus, “*F*” denotes the force generated by the stretching of the spring (equivalent to product of spring constant, *k*, and linear deformation, *x*), and “*A*” denotes the cross-sectional area of the sample prior to strain. The strain of the sample (*ε*_sample_) is quantified as the change in sample length over the original length during tensile testing. Students were provided *k* and measured *A*, *x*, and *ε*_sample_ to calculate *E*.

### Deliberate Adulterations

To facilitate learning objectives related to critical assessment of testing standards and their potential limitations, deliberate adulterations were incorporated into the experimental design. These adulterations were intended to simulate real-world discrepancies commonly encountered in testing environments and, thus, adulterations were designed to be discoverable during round-robin testing. This approach was intended to help students gain experience executing testing according to a consensus standard, while also encouraging critical thinking related to identifying and mitigating potential issues in predetermined experimental setups. The three categories of adulterations were (1) device measurement resolution, (2) device spring constant, and (3) material sourcing errors. In total, eight devices were made, two without adulterations and six each with one adulteration each. A summary of the devices and adulterations are provided in Table 1.

**Table 1:** List of device characteristics by prescribed adulteration state

Device and team numbers	Resolution	Spring constant	Material durometer	Adulteration status
1 and 2	1/16th in	1.5 lbs/in	40 A	Reference—unadulterated
3 and 4	1/4th in	1.5 lbs/in	40 A	Adulterated measurement resolution
5 and 6	1/16th in	1.0 lbs/in	40 A	Adulterated spring constant
7 and 8	1/16th in	1.5 lbs/in	60 A	Adulterated material

#### Device Measurement Resolution Adulteration

The reference demarcation on the acrylic etched ruler was 1/16th-inch resolution markings, but two devices featured an adulterated 1/4th-inch resolution markings, making precise measurement of displacement more challenging. This adulteration forced students to address how measurement errors could impact their calculations, ultimately affecting the determined elastic modulus.

#### Device Spring Constant Adulteration

Adulteration of the spring component was introduced to demonstrate the consequences of using poorly calibrated testing devices. The spring constant of the reference spring was 1.5lb/in, and the spring constant of the adulterated spring was 1.0 lbs/in. This subtle variation introduced differences in force–displacement behavior, leading to discrepancies in calculated stress and strain values. This adulteration forces students to accurately verify properties of components before conducting testing. Notably, the mock standard specified a spring constant of 1.5 lbs/in.

#### Material Sourcing Error Adulteration

Adulterations related to material sourcing were incorporated to highlight the potential impact of inconsistent or unreliable materials on testing outcomes. While students were provided with the same dumbbell die-cut shapes, the durometer ratings were either 40A or 60A. Fake companies were assigned to each set of materials with the intent of making the material sourcing error discoverable. “Techsheet Solutions” was assigned to the 40A durometer material and “Prime Materials” was assigned to the 60A durometer material. Six groups received 40A (Techsheet Solutions) dumbbells which served as the reference material, while two groups received 60A (Prime Materials) dumbbells which served as the adulterated samples. Each group was provided a bag of 5 samples with a label denoting polyurethane rubber as the material type and their respective vendor name shown. Importantly, the durometer was not disclosed to students.

### Classroom Implementation

A total of 28 students participated in the module, divided into eight teams of three to four students each. Teams were formed based on seating arrangement at the start of the module. Each team was assigned a unique table number from 1 to 8 and provided the corresponding tensile testing device according to Table 1. Students were provided with the mock testing standard, ASTM BME410-24, ahead of class to read and interpret independently. A two-day activity was originally planned for the curriculum module (75-min duration Tuesday/Thursday). On the first day of the module, students were provided with one printed copy of the mock standard, one printed activity worksheet per group, one bag of labeled vendor samples per “Material Sourcing Error Adulteration” Sect., and one custom tensile testing device. Students were given no other information with regard to the device or their adulterations and were instructed to use the tensile testing system to calculate Young’s Modulus using the mock standard, the worksheet, and their provided materials. Day one of the module was allotted for team alignment on standard interpretation, collecting information regarding the tensile device setup, recording material characteristics, and tensile testing of the material samples. Day two of the module was allotted for additional material testing and preliminary calculations. Teams organized their data collection using the module worksheet provided on day one. The last 15 min of day two were reserved for the groups to outwardly share the results of their elastic modulus calculations, where teams should ultimately learn that each group had calculated different elastic modulus values. The instructors then informed the students that at least three deliberate sources of error were introduced into the devices and each device, except for two references devices, had one adulteration only. As part of round-robin testing, students were encouraged to discuss the discrepancy between their results and to interrogate their devices and materials to eventually propose sources of adulteration. Following this, the next deliverable for the module was introduced, the Standards Revision Report, and students were allowed to observe other teams’ devices. Due to time limitations presented by the regular class times, the devices were made available to students for an additional two days (optional days three and four) if additional testing, data review, device interrogation, or discussion time was desired.

#### Tensile Testing Worksheet

The worksheet (Supplementary Materials Document 2) was used to guide students through the tensile testing process and served as a template for data collection. This worksheet facilitated the documentation of device specifications, material sample dimensions, and relevant measurements (e.g., force, displacement, stress, strain). The worksheet also required students to calculate the elastic modulus for each trial and reflect on the error in repeated measurements. An example of a completed tensile testing worksheet is provided as Supplementary Materials Document 3.

#### Round-Robin and Standard Revision Report

At the end of the module, teams submitted a Standard Revision Report, in which they (1) summarized elements of the ASTM BME410(24) standard relevant to their own testing, (2) presented their data, (3) identified what they considered to be the three device adulterations, and (4) proposed modifications to the standard to eliminate the suspected error due to adulterations. Items 1 and 4 were scored by the authors of the study for evaluation of meeting learning objectives. Item 3 is related to learning objective 2, but was not formally used in its assessment.

The summary of elements from the ASTM BME410(24) important to the team’s own tensile testing (item 1 from the report; assessment for learning objective 2) was scored to the following categories: preparation for the testing, testing performance, and evaluation of testing. For the preparation category, students were expected to at least mention the sample dogbone preparation and the collection/use of relevant equipment to perform testing. For the testing category, students were expected to at least mention loading the dogbone sample and some procedure for straining the sample. For the evaluation category, students were expected to at least mention data acquisition and the calculation of Young’s modulus. Each category was scored with discrete points of 0 (missing or neither correct requirement), 0.5 (one correct requirement), or 1 (both correct requirements).

The identification of adulterations to the tensile testing devices (item 3 from the report) was scored to the following categories: identification of the device measurement resolution adulteration, identification of the spring constant adulteration, and identification of the material adulteration. Again, each category was scored with discrete points of 0 (incomplete or missing the identification), 0.5 (partial identification), or 1 (complete identification). Partial credit corresponds to the general source of the adulteration being identified but was not completely accurate (e.g., material issue but not designated a supplier or durometer issue).

The modification to the standard to eliminate suspected error (item 4 from the report; assessment for learning objective 3) was scored to the following categories: one alteration to the ASTM BME410(24) standard and one new section to the standard. For both categories, students were expected to demonstrate consistency between their identified adulteration (item 3 of report) and their proposed modifications, and to demonstrate quality/thoroughness in their work. Again, the categories were scored with discrete points of 0 (missing, or the work was inconsistent with identified adulterations and did not demonstrate quality/thoroughness), 0.5 (either the work was consistent with the identified adulterations or it demonstrated quality/thoroughness), or 1 (the work was both consistent with the identified adulterations and it demonstrated quality/thoroughness). This scoring methodology independently accommodates quality of work and accuracy of work, both of which we believe to be necessary for technical writing.

#### Post-module Survey

A post-module survey was administered to students to solicit feedback regarding their experience during the activity. The survey consisted of ten five-point Likert scale questions and four open-ended questions. The survey assessed the students’ perception of the activity’s effectiveness in preparing them to be able to identify and apply standards in both specific and general use cases. Likert scale responses were coded to values of 1–5 for analysis, with coding provided in Table 2. University-licensed Microsoft Copilot was used to compile the responses to the open-ended questions in the post-survey. For each open-ended question, the responses were copied and pasted into Microsoft Copilot paired with the following prompt: “Given the following responses, summarize the student’s feedback for the question provided in 3 bullet points, limited to one sentence each, highlighting the common themes or similarities in the responses making sure to not quote any one response directly.” All Microsoft Copilot generated responses were verified by the authors for accuracy and authenticity to the student’s responses. For assessment of learning objective 1 (the definition of round-robin testing), the first open-ended response was also scored by the authors of the study according to the inclusion of two principal components: the purpose of round-robin testing being reproducibility, and that testing is conducted independently by multiple participants. Definitions were scored with discrete points of 0 (missing or neither correct component), 0.5 (one correct component), or 1 (both correct components).

**Table 2:** Likert scale response codes for the post-module survey

Question number	1	2	3	4	5
1–7	Very unconfident	Somewhat unconfident	Not sure	Somewhat confident	Very confident
8	Very negatively impacted	Somewhat negatively impacted	Not sure	Somewhat positively impacted	Very positively impacted
9	Very ineffective	Somewhat ineffective	Not sure	Somewhat effective	Strongly effective
10	Very disagreeable	Somewhat disagreeable	Not sure	Somewhat enjoyable	Very enjoyable

## Results

The results present the scoring of the Standard Revision Report and findings from the post-module survey.

### Standard Revision Report: Summary of Elements from ASTM BME410(24)

Table 3 presents scoring from the Standard Revision Report regarding the summary of elements from the ASTM BME410(24) mock standard that students determined were useful for their own tensile testing. Teams were scored according to the identification and summary of key aspects to tensile testing. Briefly, six teams, at least in part, correctly summarized all three scored categories. Every team, in part, accurately summarized the preparation for testing and testing performance categories. The evaluation of testing category had the lowest scoring and was summarized, at least in part, by six of the eight teams.

**Table 3:** Scoring of the Standard Revision Report for summary of elements from the ASTM BME410(24) mock standard that were useful for customized tensile testing

Device and team number	Adulteration	Summary of preparation for testing	Summary of testing performance	Summary of evaluation of testing	Total summary score
1	None	1	1	1	3
2	None	1	1	0	2
3	Resolution	1	1	0	2
4	Resolution	1	1	1	3
5	Spring	1	1	0.5	2.5
6	Spring	1	0.5	0.5	2
7	Sourcing	0.5	1	1	2.5
8	Sourcing	1	1	1	3
		0.9 ± 0.2	0.9 ± 0.2	0.6 ± 0.4	2.5 ± 0.5

Scores for the categories were as follows: 0 = missing or neither correct requirement, 0.5 = one correct requirement, or 1 = both correct requirements. An average and standard deviation for each category and the total score are provided

### Standard Revision Report: Identification of Adulterations

Table 4 presents the results from the Standard Revision Report which scores group performance in identifying each of the adulterations present in the tensile testing devices during a round-robin activity. Briefly, three teams identified, at least in part, all three adulterations. Three teams identified the measurement resolution adulteration, whereas six identified the spring constant adulteration and four identified the material source adulteration. Notably, no team fully identified the material source adulteration.

**Table 4:** Scoring of the Standard Revision Report for identification of the alterations to the tensile testing devices

Device and team number	Adulteration	Identification of resolution adulteration	Identification of spring adulteration	Identification of material adulteration	Total adulteration identification score
1	None	1	1	0.5	2.5
2	None	0	0	0	0
3	Resolution	1	1	0.5	2.5
4	Resolution	0	0	0	0
5	Spring	0	0.5	0	0.5
6	Spring	0	0.5	0	0.5
7	Sourcing	1	1	0.5	2.5
8	Sourcing	0	1	0.5	1.5
		0.4 ± 0.5	0.6 ± 0.4	0.3 ± 0.3	1.3 ± 1.1

Scores were as follows: 0 = not identified, 0.5 = partial identification, and 1 = complete identification. An average and standard deviation for each adulteration and the total score are provided

### Standard Revision Report: Modification of the ASTM BME410(24) Standard

Table 5 presents scoring from the Standard Revision Report regarding the modification of the ASTM BME410(24) mock standard following round-robin testing. Teams were evaluated for consistency of their modification to their identified adulterations. Briefly, seven of eight teams, at least in part, altered the standard and proposed a new section of the standard to address their identified adulterations. One team failed to propose a new section of the standard.

**Table 5:** Scoring of the Standard Revision Report for modification of the ASTM BME410(24) mock standard

Device and team number	Adulteration	Alteration of standard	New section of standard	Total modification score
1	None	1	0.5	1.5
2	None	0.5	0	0.5
3	Resolution	1	1	2
4	Resolution	1	1	2
5	Spring	0.5	1	1.5
6	Spring	0.5	0.5	1
7	Sourcing	1	1	2
8	Sourcing	0.5	1	1.5
		0.8 ± 0.3	0.8 ± 0.4	1.5 ± 0.5

Scores were as follows: 0 = missing, or inconsistent with adulterations and did not demonstrate quality/thoroughness, 0.5 = consistent with adulterations or demonstrated quality/thoroughness, or 1 = consistent with adulterations and demonstrated quality/thoroughness. An average and standard deviation for each category and the total score are provided

### Post-module Survey

#### Multiple Choice Questions

**Fig. 2:**
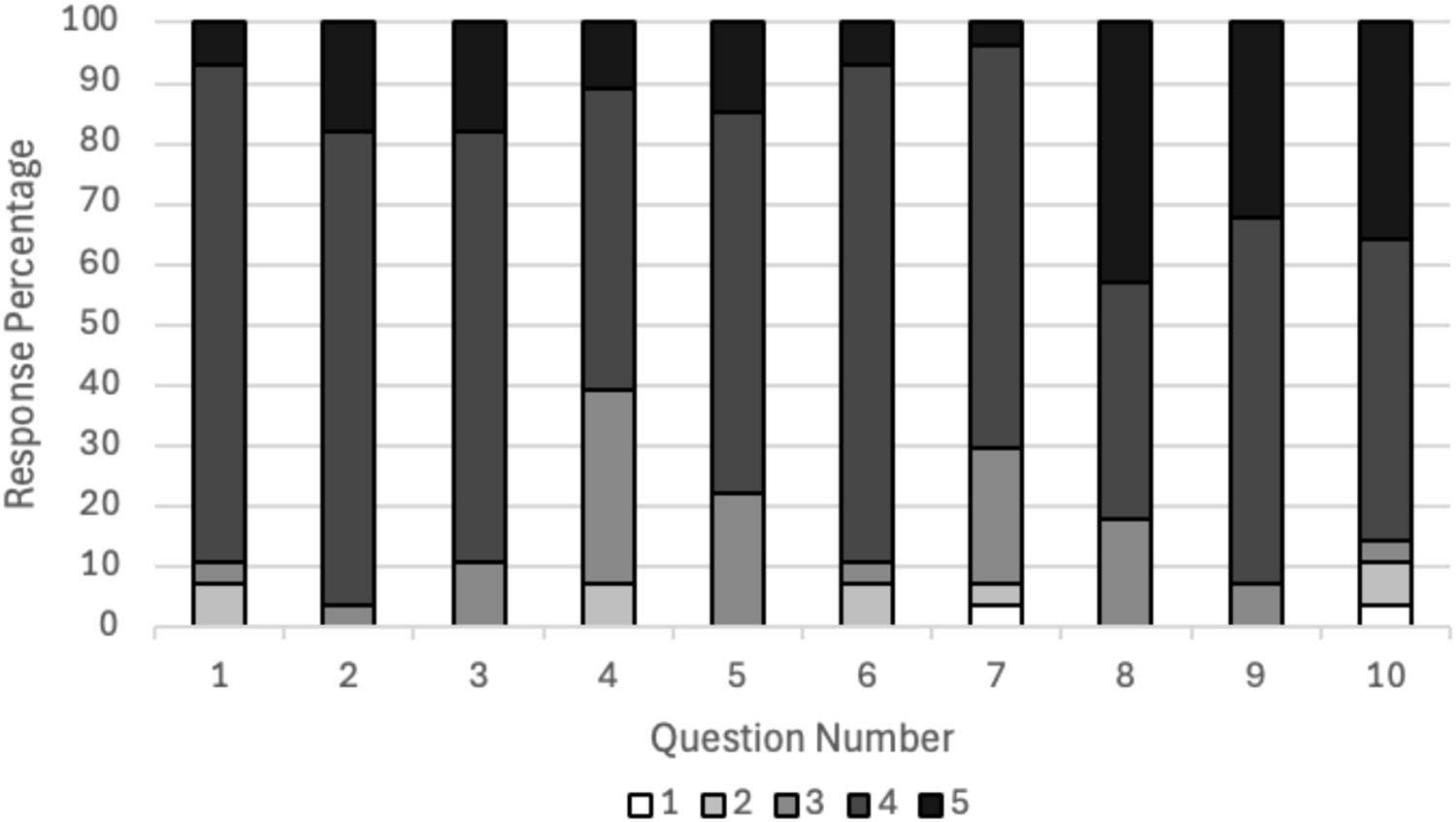
Percentage distribution of Likert Scale Responses by Question. Questions 1-7 correspond to students’ self-assessment of confidence in certain skills after completion of the module. Questions 8-10 correspond to student reflection on module. Response values range from 1-5 (1 = low; 5 = high)

The average score of the responses to survey questions are presented in Tables 6 and 7. Percentage distributions of the responses are provided in Figure 2. For Questions 5 and 7, one participant each neglected to respond. Briefly, average confidence among the various topics trended toward “somewhat confident.” The lowest confidence was reflected in the application of consensus standard in customized cases, whereas the highest was reflected in application of consensus standard to general cases and extracting requirements from a standard.

**Table 6:** Likert scale questions from student survey to assess confidence in certain skills after completion of the module (1 = Very Unconfident, 2 = Somewhat Unconfident, 3 = Not sure, 4 = Somewhat Confident, 5 = Very Confident). Average score, standard deviation, and number of responses (N) are provided

Question number	Summarized question content	Average ± Standard Deviation (N)
1	Rank your confidence in using appropriate tools to identify an appropriate consensus standard for a given technology or process	3.9 ± 0.63 (28)
2	Rank your confidence in extracting product requirements/specifications from consensus performance standards for a given technology or process	4.1 ± 0.45 (28)
3	Rank your confidence in applying consensus standards in general use cases (i.e., when a standard applies to a process or procedure)	4.1 ± 0.54 (28)
4	Rank your confidence in applying consensus standards in customized use cases (i.e., when not all of the standard applies to your concept)	3.6 ± 0.78 (28)
5	Rank your confidence in identifying potential limitations in consensus standards	3.9 ± 0.62 (27)
6	Rank your confidence in applying consensus standards for conducting verification testing	3.9 ± 0.63 (28)
7	Rank your confidence in conducting round-robin testing of consensus standards	3.6 ± 0.79 (27)

**Table 7:** Likert scale questions from student survey to reflect on the round-robin standards testing learning module (1 = low; 5 = high). Average score, standard deviation, and number of responses (N) are provided

Question Number	Summarized Question Content	Average ± Standard Deviation (N)
8	Rank your perception for how the Round-Robin Testing module helped prepare you to apply your knowledge of consensus standards in industry	4.3 ± 0.76 (28)
9	Rank how effective you found the Round-Robin Testing module in preparing you to assess a standard for use in verification testing.	4.3 ± 0.59 (28)
10	Rank your enjoyment of the Round-Robin Testing module (activity and deliverable)	4.1 ± 1.0 (28)

#### Open-Ended Questions

The responses to the four open-ended questions from the post-module survey are provided in Table 8. The responses were summarized into three succinct bullet points with brief descriptions using Microsoft Copilot. Question 12 was scored to assess learning objective 1, with a mean and standard deviation of 0.4 ± 0.5 (out of 1). Scoring distribution was virtually even between scores of 0 (N = 10), 0.5 (N = 9), and 1 (N = 8).

**Table 8:** Open-ended survey questions, number of responses per question, and respective responses as summarized into three bullet points by Microsoft CoPilot

Number	Question	Number of responses	Bullet point 1	Bullet point 2	Bullet point 3
11	What key takeaways did you learn from this module to apply to your knowledge of using consensus standards in industry?	25	Precision and Detail: Emphasizing the importance of precision in measurements and detailed understanding of standards to avoid errors and ensure accurate results.	Collaboration and Verification: Highlighting the value of collaboration and cross-laboratory verification to confirm the reliability and consistency of standards.	Thorough Understanding and Application: Stressing the need to thoroughly read and understand standards, ensuring proper application and continuous verification to maintain effectiveness.
12	Define round-robin testing of consensus standards in 1-2 sentences.	27	Consistency and Accuracy: Emphasizing the importance of verifying the consistency and accuracy of measurement methods through repeated testing.	Collaboration and Comparison: Highlighting the collaborative nature of round-robin testing, where multiple groups or laboratories compare results to identify discrepancies and improve standards.	Reliability and Reproducibility: Stressing the need for reliable and reproducible results to confirm the effectiveness and applicability of the standards in various contexts.
13	What are some key challenges and considerations for interpreting, applying, and revising consensus standards?	21	Complexity and Clarity: Understanding and interpreting the detailed and often complex language of standards, ensuring clear and precise instructions to avoid misinterpretation.	Consistency and Relevance: Maintaining consistent application of standards while keeping them relevant amid technological advancements and industry changes.	Collaboration and Consensus: Achieving agreement among diverse stakeholders and ensuring that revisions are safe, cost-effective, and aligned with legal and global requirements.
14	Provide comments or suggestions for improving the module’s focus on standards revision and testing.	18	Extended Duration and Detailed Instructions: Suggesting the module be extended to 2 weeks and providing more detailed instructions to help students grasp the concepts better.	Proper Equipment Setup: Emphasizing the need for properly setup equipment to ensure accurate testing and boost student confidence in their results.	Real-World Examples and Collaboration: Recommending the inclusion of more real-world examples and opportunities for collaboration to enhance understanding and engagement.

## Discussion

Here, we describe a curricular module for the modification of a mock consensus standard through round-robin testing to characterize the Young’s modulus of a polyurethane material using a custom tensile testing device. The purpose of the module is to enhance engineering student preparedness and understanding of engineering industry practice with consensus standards. Select testing devices were purposefully adulterated to facilitate differential outcomes from the module. This module was piloted as part of a major course update to our FDA and ISO Standards for the Design and Manufacture of Medical devices course (BME 410). Student teams actively identified relevant parts of the mock standard to perform, characterized the Young’s modulus of their material, and effectively applied round-robin methodology to identify sources of error between teams. After round-robin testing, teams authored a Standard Revision Report to propose modifications to the mock standard to eliminate sources of potential error. Students also completed a post-module survey to assess their understanding of module content. The learning objectives of the module were formulated to span all of Bloom’s Taxonomy. Learning objectives of the module included the following: (1) define round-robin standards assessment process (levels 1 and 2), (2) interpret and apply a consensus standard (levels 3 and 4), and (3) revise a consensus standard (levels 5 and 6).

The first learning objective was assessed using the individual definitions of round-robin testing from the post-module survey. The Microsoft Copilot summary of round-robin testing definitions indicated themes of consistency, accuracy, collaboration, comparison, reliability, and reproducibility. These themes are consistent with appropriate definitions of round-robin testing. However, the definitions were scored at 0.4 ± 0.5 (out of 1), corresponding to an average of 40%. Thus, on average, students could supply approximately half of the expected definition. The discrepancy between the Microsoft Copilot assessment and our own may be attributable to our searching for exact components, which is less flexible than an overall summary. Nevertheless, 17/28 students (63%) could supply at least partial definition, and including the more holistic assessment from Microsoft Copilot, we feel confident the students reasonably met the first learning objective.

The second learning objective was assessed using the summary of elements from the mock standard that students determined were useful for their own tensile testing. From the composite scores of the summaries, the mean and standard deviation were 2.5 ± 0.5 (out of 3), corresponding to an average score of 83%. From this assessment, all teams but one scored full points in the preparation and testing performance categories. The evaluation of testing was the lowest scored category, with two teams scoring no points. We suspect this discrepancy in scoring between categories is related to the ability of the teams to identify relevant elements from the mock standard that immediately reflected what they had in front of them (i.e., preparation and testing reflecting the device and materials they received) rather than what would be required after testing (i.e., evaluation of performance). From these data, we feel confident the students met the second learning objective. Identification of correct adulterations was not required for the activity to be efficacious and for students to meet learning objectives. Instead, we considered consistency between identified adulterations (even if incorrect) and the revisions to the mock standard to be more important. Nevertheless, it was useful to assess the ability of teams to correctly identify adulterations as it provides an opportunity to reflect on their relative difficulty to identify (primarily a pragmatic consideration for the sake of learning in this module), which may drive changes to the adulteration selections in future module implementations. From the standards revision report, six of eight teams (75%), at least in part, identified an adulteration, with three teams scoring 2.5/3. Though, the mean and standard deviation adulteration score was 1.3 ± 1.1 (out of 3), corresponding to an average of 42%. Of the adulteration sources, the adulterated spring constant was seemingly the easiest for groups to identify, while the material adulteration proved to be the most challenging. These results suggest that while students demonstrated a reasonable ability to detect adulterations in general, their ability to correctly identify an adulteration varied based on the adulteration type and the group’s interpretation of the provided standard. Notably, each of the three teams that scored 2.5/3 identified an issue with the supplied material. Specifically, they observed that some material sets had striations perpendicular to others suggesting an anisotropy of the material. As such, these were not incorrect observations, but the teams did not easily identify that they were labeled as being manufactured by different vendors. This variability in group performance, as reflected in the differing scores for adulteration identification, underscores the need for more structured guidance during the early stages of the module, such as guidance of more detailed notes on the worksheet, to ensure a uniform understanding of expected testing outcomes and critical evaluation techniques. Moreover, different adulterations to the devices may be considered to enhance performance and facilitate the connection between adulteration and standard revision.

The third learning objective was assessed using the modifications made to the standard. From the composite modification scores, the mean and standard deviation were 1.5 ± 0.5 (out of 2), corresponding to an average score of 75%. All teams scored points in the alteration category, and all but one team scored points in the new section category. These results indicate general consistency and quality in the modification of the mock standard. Notably, this section was scored according to the team’s identified adulterations, so even if they did not accurately identify an adulteration, it did not preclude quality work in the modification of the standard.

Though most of it was not assessed as part of the learning objectives, the post-module survey was an important opportunity for us to reflect on the efficacy of the module in general as well as collect useful feedback about its implementation. Analysis of the post-module survey Likert Scale responses indicates a generally positive reception of the module, with students expressing moderate to high confidence in their ability to apply consensus standards. In particular, students reported higher confidence in extracting product requirements and applying standards to general use cases compared to customized use cases. Here, we probed the understanding of following a standard “verbatim” (i.e., in the general case with a device that one would claim full compliance with the standard) compared to customized use cases where one must identify appropriate portions of a standard which require compliance. The lower confidence in customized use cases suggests that additional instructional time or supplementary materials might be necessary to build advanced skills in adapting standards to unique contexts. The open-ended question portion of the survey offered an opportunity for capturing critical feedback and responses highlighted the importance of precision, collaboration, and reproducibility when applying consensus standards. These responses mirrored those to the Likert scale questions and students shared that the module could be enhanced by providing more time and detailed instructions.

There are several limitations to the current study. First, the sample size was relatively small with only 28 students across eight teams, which may limit the characterization of the study’s findings. Additionally, the module was implemented within a single BME course and further research is necessary to assess its applicability across diverse engineering disciplines and institutions. Second, there was a wide scoring and performance distribution among teams, particularly in the ability to identify adulterations. The teams that scored higher overall (teams 1, 7, and 8, compared to teams 2 and 6) were those that also attended the two optional days, highlighting the importance of participation and collaboration during the activity to maximize learning outcomes. Anecdotally, the higher scoring teams were typically composed of higher performing students who self-assembled into teams via their seating proximity. In contrast, some teams that scored lower misinterpreted the goals of the activity and did not leverage the opportunity for collaboration on the two additional testing days. Deliberate team assignments in future module implementations may help to avoid large deviations in performance between teams. Third, a pre-module survey was not administered. This would be useful to evaluate the differential responses between the pre- and post-module survey and assess the effects of the module. Lastly, there was no capacity to evaluate the effect of the module on the careers of the students. Future studies to explore long-term impacts of the module beyond the course (e.g., in industry roles) would be useful to gauge efficacy and serve as an opportunity for respondents to reflect on the feedback they originally provided.

As a result of these limitations and our findings, there are several general modifications and considerations for future implementation. First, integration of consensus standards across engineering curricula improves fundamental understanding of consensus standards as observed by Rooney et al. [11]. Second, within the module, students may benefit from extended duration (e.g., increasing from structured 2- to 4-day activity) and more explicit instructions (e.g., specifying to review supplier information, explicitly sharing which sets of tensile testing machines were adulterated similarly), consistent with student feedback. Third, integrating additional complex tasks aimed at higher levels of Bloom’s Taxonomy (e.g., writing a new standard without predicate, a consensus standard draft from the entire class) could further enhance student capabilities in customized and innovative applications of standards. To fully address student open-ended comments, it would likely be helpful to provide better guidance in loading samples and setting up the tensile testing system while also providing more context for why elastic modulus calculations are important.

A module for round-robin testing and revision of a consensus standard was introduced. This work not only reinforces fundamental engineering principles but also prepares students for the complex and regulated environment of the medical device industry. Though this module was designed to enhance BME education, it is scalable to any engineering discipline that benefits from materials characterization and utilizes consensus standards.

## Supplementary Information

Below is the link to the electronic supplementary material.

Supplementary file1 (XLSX 15 KB)

Supplementary file2 (Docx 36 KB)

Supplementary file3 (Docx 822 KB)

## Data Availability

All materials are made available at the module repository. Data are available upon reasonable request to the authors.
