# Supplementary material for: Developing a Round-Robin Module For The Integration Of Consensus Standards In a BME Course Using a Custom Tensile Testing Device
Source: Biomed Eng Educ. 2025 Oct 7;6(1):83–95. doi: 10.1007/s43683-025-00200-x (PMC12876107; doi:10.1007/s43683-025-00200-x)
Supplement: Supplementary file 3 — Supplementary file3 (Docx 822 KB) [file 43683_2025_200_MOESM3_ESM.docx]

**Tensile Testing Activity Worksheet**


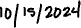

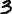


List the names of all team members:

Start Time:

End Time:

     - Name 1: _________________________________

     - Name 2: _________________________________

     - Name 3: _________________________________

     - Name 4: _________________________________

- Name 5: _________________________________

**Instructions:**

This worksheet accompanies the activity, which involves calculating the elastic modulus of materials using custom tensile testing devices in compliance with ASTM BME410-24. Please follow the instructions carefully and fill out each section with your observations.

1. Tensile Testing Device Setup: Record specifications based on ASTM BME410-24. Inspect your device; note any characteristics critical to calculating elastic modulus (e.g. spring constant, measurement resolution, gear tooth-count, length of webbing). Include units.

____________________________________________________________________________________


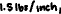

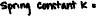

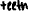

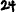


____________________________________________________________________________________


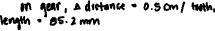

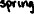


____________________________________________________________________________________

2. Sample Identification: Record the material source and dog bone sample dimensions based on ASTM BME410-24. Include units.

Vendor: ____________________________________________________________________________


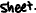

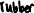

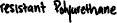

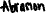

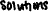

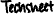


| Sample | Gauge length | Thickness | Width |
| --- | --- | --- | --- |
| A | 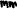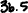 | 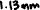 | 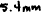 |
| B | 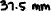 | 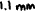 | 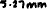 |
| C | 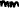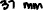 |  |  |
| D | 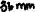 | 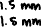 | 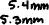 |
| E | 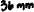 | 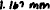 | 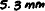 |

Notes:

____________________________________________________________________________________

____________________________________________________________________________________

____________________________________________________________________________________

____________________________________________________________________________________

____________________________________________________________________________________

____________________________________________________________________________________

**Data Collection – Standard Sample Testing:**


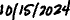

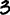


For each test, you will use a tensile testing device and record the applied force, displacement, and any other relevant measurements. Follow the “ASTM D410-24” standard procedures and include units for all measurements.


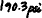


|  | Measured | | Calculated | | | |
| --- | --- | --- | --- | --- | --- | --- |
| Sample (Trial) | Material Displacement | Spring displacement | Applied Force | Material Stress | Material Strain | Elastic Modulus |
| A (1) | 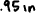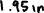 | 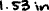 | 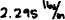 | 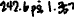 |  |  |
| A (2) | 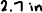 | 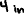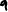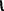 |  | 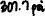 | 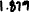 | 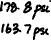 |
| A (3) | 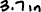 | 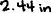 | 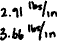 | 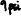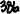 | 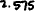 | 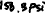 |
|  |  |  |  |  |  |  |
| B (1) | 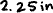 | 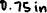 | 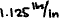 | 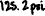 | 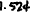 | 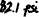 |
| B (2) | 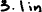 | 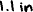 |  | 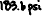 | 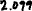 | 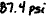 |
| B (3) | 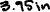 | 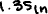 | 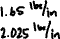 | 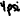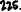 | 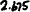 | 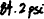 |
|  |  |  |  |  |  |  |
| C (1) | 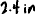 | 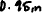 |  | 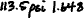 |  | 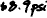 |
| C (2) | 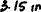 | 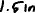 | 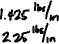 |  | 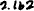 | 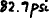 |
| C (3) | 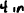 | 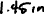 | 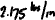 | 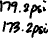 | 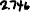 | 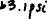 |
|  |  |  |  |  |  |  |
| D (1) | 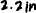 | 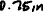 | 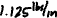 | 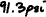 | 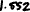 | 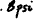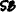 |
| D (2) | 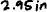 | 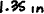 |  | 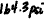 | 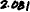 | 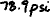 |
| D (3) | 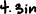 | 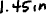 | 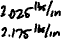 |  |  |  |
|  |  |  |  |  |  |  |
| E (1) |  |  |  |  |  |  |
| E (2) |  |  |  |  |  |  |
| E (3) |  |  |  |  |  |  |
|  |  |  |  |  |  |  |
| **AVERAGE** |  |  |  |  |  |  |
